# From Endometriosis to Lipedema: Toward a Neuroimmune Framework for Pain Amplification in Hormone-Sensitive Disorders

**DOI:** 10.3390/biomedicines14071510

**Published:** 2026-07-03

**Authors:** Diogo Pinto da Costa Viana, Thiago Bracks Oliveira, Adriana Luckow Invitti, Eduardo Schor

**Affiliations:** 1 Department of Gynecology, Escola Paulista de Medicina, Federal University of Sao Paulo (EPM-UNIFESP), Sao Paulo 04024-002, Brazil; 2 Brazilian Society for Research and Teaching in Medicine (SOBRAPEM), Sao Paulo 01318-901, Brazil

**Keywords:** endometriosis, lipedema, neuroimmunomodulation, chronic pain, calcitonin gene-related peptide, mast cells, neurogenic inflammation, central nervous system sensitization

## Abstract

**Background**: Endometriosis and lipedema are chronic female-predominant disorders characterized by persistent pain that is frequently disproportionate to anatomical lesion burden. Although traditionally interpreted within distinct lesion-centered frameworks, both conditions exhibit striking clinical and epidemiological parallels, including hormonally modulated symptom dynamics, overlap with central pain syndromes, weak correlation between structural disease severity and pain intensity, and symptom clustering during reproductive transitions such as puberty, pregnancy, and menopause. **Methods**: This study aims to synthesize clinical, molecular, neuroimmune, and endocrine evidence on the interrelationship between endometriosis and lipedema, and to propose a hypothesis-generating neuroimmune framework linking both conditions. This integrative narrative review conducted a non-systematic literature search in PubMed/MEDLINE, Scopus, and Web of Science, focusing on mechanisms related to chronic pain, mast cell biology, TRPV1 signaling, CGRP-mediated neurogenic inflammation, intracrine steroidogenesis, and peripheral and central sensitization. **Results**: The review identifies convergent biological characteristics between the two diseases, including mast cell activation, macrophage polarization, endothelial dysfunction, fibrosis, angiogenesis, intracrine estrogen metabolism, and persistent inflammatory signaling. In endometriosis, direct evidence demonstrates increased sensory innervation, nerve growth factor expression, TRPV1 sensitization, CGRP-positive fibers, and mast cell-nerve interactions. In lipedema, convergent upstream mechanisms, including mast cell infiltration, elevated histamine levels, adipose tissue inflammation, and local estrogen activation, support the plausibility of a functionally analogous neuroimmune organization, despite incomplete direct neural characterization. In this context, the mast cell-TRPV1-CGRP axis is proposed as a biologically plausible framework, directly supported in endometriosis and currently hypothetical in lipedema, connecting peripheral sensitization, neurogenic inflammation, hormonal chronodependence, and central nociceptive amplification. The model further conceptualizes pain crises as transient events of instability within a sensitized neuroimmune network and proposes mechanistic phenotypes that integrate gastrointestinal, inflammatory, central, and hormonal triggers. **Conclusion:** Endometriosis and lipedema may represent topographically distinct manifestations of a shared neuroimmune process operating within hormone-sensitive tissues. Although the evidentiary basis remains asymmetric, with stronger mechanistic support in endometriosis than in lipedema, this framework provides a biologically plausible and experimentally testable model integrating endocrine, immune, neural, and vascular contributors to chronic pain amplification. This perspective supports coordinated translational investigation across reproductive biology, endocrinology, and pain medicine and may contribute to future mechanism-based stratification and therapeutic development. This work is hypothesis-generating and is not intended to establish causality or to provide clinical recommendations; all proposed mechanistic and therapeutic inferences require prospective experimental validation.

## 1. Introduction

Chronic pain that is disproportionate and refractory is a major clinical burden in two hormone-responsive disorders that predominantly affect women: endometriosis and lipedema, both historically interpreted within lesion-centered frameworks. Endometriosis, an estrogen-dependent chronic inflammatory disease defined by endometrium-like tissue outside the uterine cavity [1,2], affects approximately 10% of women of reproductive age and causes progressive dysmenorrhea, chronic pelvic pain, deep dyspareunia, and infertility [1]. Lipedema is a disproportionate, bilateral, symmetrical expansion of subcutaneous adipose tissue, predominantly in the limbs, affecting up to 11% of adult women, with spontaneous pain, pressure tenderness, easy bruising, and dermal hypersensitivity that frequently precedes and persists independently of tissue expansion [3,4,5,6].

Despite distinct anatomical substrates and historical separation across specialties, both share clinical features not previously unified. The most consistent is the dissociation between anatomical lesion burden and pain intensity. In endometriosis, the American Society for Reproductive Medicine (ASRM) staging system correlates weakly with symptom severity, and many patients have persistent pelvic pain after anatomically adequate resection [7,8,9,10]. In lipedema, pain does not scale with disease stage or adipose volume, and the benefit of tumescent liposuction is often not sustained [3,4,11]. In both, episodic exacerbations occur under stable hormonal conditions without detectable lesional progression [12,13,14].

This dissociation suggests that the anatomical substrate acts primarily as an initiator of nociceptive signaling rather than its exclusive driver.

Comorbidity profiles overlap further: endometriosis with migraine, IBS, painful bladder syndrome, and vulvodynia [7,15,16,17,18]; lipedema with fibromyalgia (≈35%), anxiety, depression, headache, and gastrointestinal disorders [13,19,20]. Both may represent distinct peripheral expressions of a broader nociceptive vulnerability.

Temporal alignment is also striking. Both are hormonally modulated, with onset or exacerbation at puberty, pregnancy, and menopause [1,6,14]. In lipedema, onset or worsening has been reported in 62–72% of cases at puberty, 53% during pregnancy, and 67.9% at menopause, paralleling endometriosis; these figures derive from a single scoping review [14] and require independent replication.

A recent scoping review described consistent clustering of lipedema with gynecologic and endocrine conditions, including endometriosis, across the included cohorts [14]. As this evidence derives from a single synthesis, the association should be regarded as preliminary and requiring independent confirmation, while still warranting mechanistic explanation.

We propose a hypothesis-generating framework in which endometriosis and lipedema are conceptualized as topographically distinct expressions of a shared neuroimmune axis: a steroid-responsive tissue (ectopic endometrium or subcutaneous adipose tissue) undergoes local estrogenic reprogramming into an algogenic microenvironment of coordinated sensory afferents, mast cells, polarized macrophages, and microvasculature, generating sustained peripheral and central nociceptive amplification.

This hypothesis rests on convergent, though asymmetric, molecular and histological evidence. In endometriosis, lesions show increased sensory nerve density, elevated NGF, upregulation and sensitization of TRPV1, mast cell infiltration near nociceptive afferents, CGRP release, local aromatase-driven estradiol biosynthesis, and macrophage polarization toward inflammatory and profibrotic phenotypes [2,21,22,23,24,25,26,27,28]. In lipedema, subcutaneous adipose tissue shows a partially analogous architecture: adipocyte hypertrophy, crown-like macrophage structures, CD163^+^ M2-polarized predominance, mast cell infiltration with elevated histamine, endothelial dysfunction, and intracrine estrogen metabolism [3,29,30,31,32,33,34,35].

**Figure 1:**
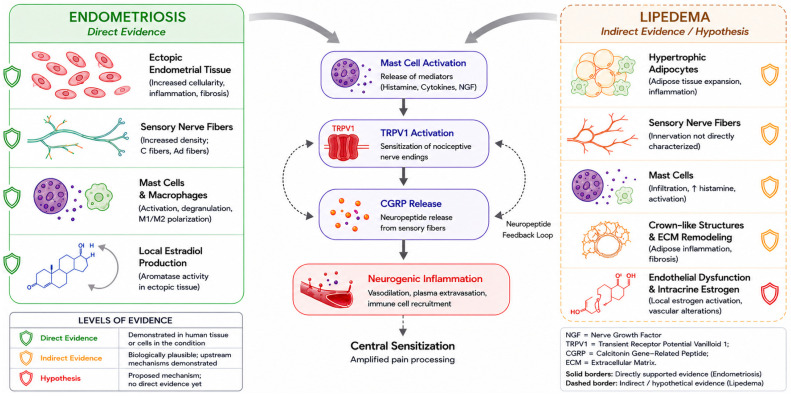
Neuroimmune axis in hormone-sensitive tissues. Endometriosis and lipedema are represented as distinct tissue contexts converging on a shared pathway in which mast cell activation promotes TRPV1 sensitization and CGRP release, leading to neurogenic inflammation and central sensitization. Solid elements indicate mechanisms supported by direct evidence in endometriosis, whereas dashed elements represent pathways that are biologically plausible but not yet directly demonstrated in lipedema. Legend: TRPV1: transient receptor potential vanilloid 1; CGRP: calcitonin gene-related peptide.

Although direct neural characterization in lipedema remains incomplete, convergence of upstream sensitizing mechanisms and functional nociceptive evidence [36] supports a functionally convergent, though structurally asymmetric, neuroimmune organization. Within this context, the mast cell-TRPV1-CGRP axis is proposed as a biologically plausible integrative framework (Figure 1), supported by direct evidence in endometriosis and by convergent upstream evidence in lipedema, linking local inflammation to sustained nociceptive amplification [37,38,39,40,41]. This axis provides a biologically plausible and integrative framework for explaining key shared features, including disproportionate pain, persistence after anatomical control, hormonally modulated exacerbations, and overlap with central pain syndromes [16,19,38,41].

On the basis of these clinical, epidemiological, and biological observations, the present review is built around four primary hypotheses, which are addressed sequentially in the following sections:

**H1.**  *Endometriosis and lipedema share a common neuroimmune organization of hormone-sensitive tissues, in which mast cells, polarized macrophages, the microvascular compartment, and sensory afferents are coordinately reorganized into an algogenic microenvironment*.

**H2.**  *Within this microenvironment, the mast cell-TRPV1-CGRP axis functions as a convergent integrative node linking local inflammation, neurogenic signaling, and central nociceptive amplification*.

**H3.**  *Intracrine steroidogenesis, particularly aromatase-mediated local estradiol biosynthesis, defines a state of hormonal chronodependence that explains the temporal clustering of symptoms across puberty, pregnancy, and menopause and the asymmetric behavior of both diseases at menopause*.

**H4.**  *Pain flares represent transient excursions of a sensitized, high-gain neuroimmune network beyond its stability margin, classifiable by their primary system of entry (gastrointestinal, central, systemic inflammatory, or hormonal) rather than by anatomical pain location*.

These hypotheses are not mutually exclusive and are tested throughout this review against existing molecular, histological, neurophysiological, and clinical evidence, with explicit acknowledgement of the asymmetry between directly demonstrated mechanisms in endometriosis and convergent but indirect evidence in lipedema.

## 2. Methods

### 2.1. Study Design

This study was designed as a narrative integrative review with a hypothesis-generating purpose. Its aim was to synthesize clinical, molecular, neuroimmune, and endocrine evidence regarding the interrelationship between endometriosis and lipedema, and to develop an integrated mechanistic framework. The review was structured and reported in accordance with the Scale for the Assessment of Narrative Review Articles (SANRA) [42], covering: explanation of importance, statement of aims, reproducibility of the literature search, referencing and citation, evidence grading, and presentation of authors’ interpretation.

### 2.2. Search Strategy

**Figure 2:**
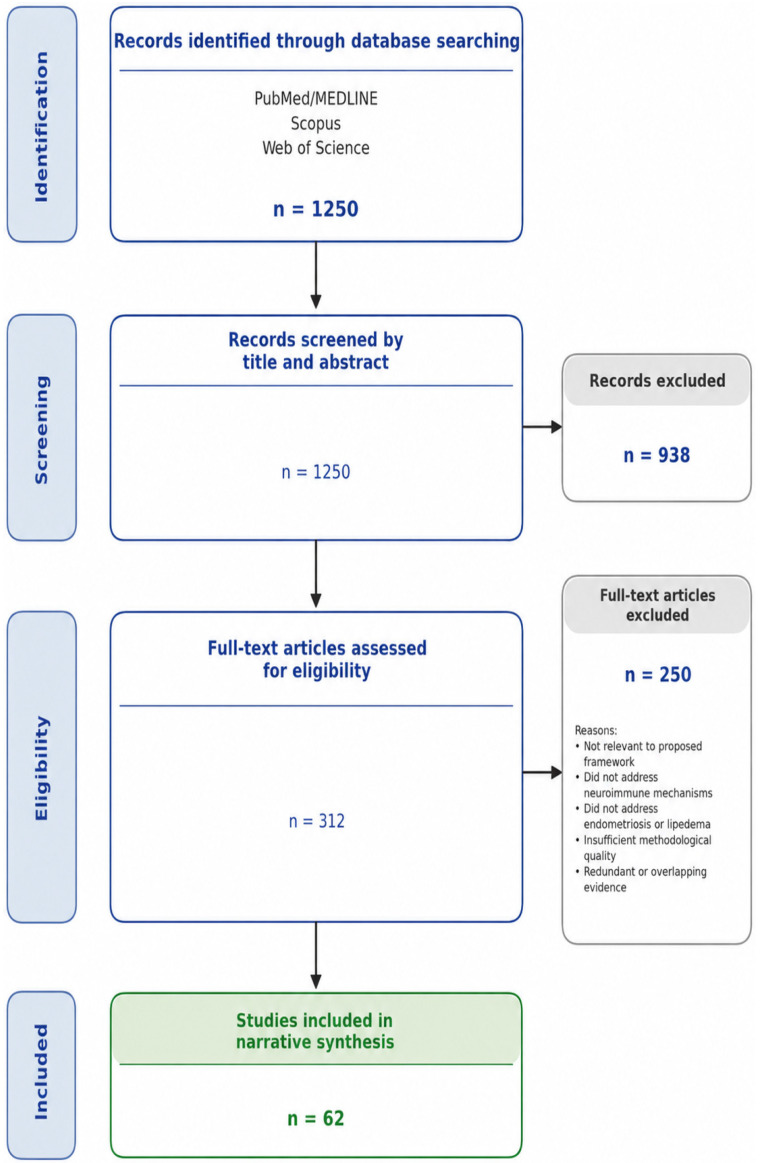
Literature search and study selection process. A structured, non-systematic search of PubMed/MEDLINE, Scopus, and Web of Science yielded approximately 1250 unique records, all screened by title and abstract (938 excluded). Of 312 full-text articles assessed for eligibility, 250 were excluded for the reasons shown, and 62 studies were included in the narrative synthesis based on conceptual relevance, methodological adequacy, and contribution to the proposed neuroimmune framework. This flow diagram is provided for transparency and does not imply a systematic review methodology.

A structured, non-systematic literature search was performed in PubMed/MEDLINE, Scopus, and Web of Science between January 2024 and February 2026. Boolean combinations of MeSH and free-text descriptors were used. The full search expression was: (“endometriosis” OR “lipedema” OR “lipoedema”) AND (“chronic pain” OR “pelvic pain” OR “nociplastic pain” OR “central sensitization” OR “peripheral sensitization”) AND (“mast cell*” OR “TRPV1” OR “CGRP” OR “neurogenic inflammation” OR “nerve growth factor”) AND (“estrogen*” OR “aromatase” OR “intracrine” OR “hormonal modulation”). Searches were limited to English-language records and to peer-reviewed publications. Approximately 1250 unique records were screened by title and abstract, of which 312 were retrieved in full text and 62 were selected on the basis of conceptual relevance, methodological adequacy, and contribution to the proposed framework. The complete literature search and study selection process is summarized in Figure 2, which is provided for transparency and does not imply a systematic review methodology.

The search was conducted iteratively and complemented by manual screening of the reference lists of included studies (snowballing), with additional records identified through PubMed’s “similar articles” feature. Disagreements regarding inclusion were resolved by consensus among authors.

### 2.3. Eligibility Criteria

Studies providing clinical, epidemiological, molecular, histological, neurophysiological, or translational evidence relevant to pain mechanisms, inflammation, hormonal regulation, and neuroimmune interactions in endometriosis and/or lipedema were included. Original research articles, systematic and narrative reviews, meta-analyses, and high-quality preclinical studies in human-relevant models were considered eligible.

No restrictions were applied regarding study design. Selection prioritized recent (2018–2026) primary studies for direct mechanistic evidence, seminal earlier studies whose findings remain authoritative, and studies addressing convergent mechanisms across both diseases. Studies limited to non-mammalian models, case reports without mechanistic implications, or non-peer-reviewed sources were excluded; conceptual relevance, biological plausibility, and contribution to the integrative framework guided final selection.

### 2.4. Data Synthesis

Data were analyzed using a narrative interpretative approach across seven mechanistic domains: tissue microenvironment, peripheral sensitization, mast cell-nerve interaction, central sensitization, hormonal chronodependence and intracrine steroidogenesis, network instability and pain flares, and translational implications. Each domain was evaluated against a structured evidence-asymmetry matrix (direct evidence in endometriosis/inferred in lipedema/shared upstream mechanism) to identify convergent patterns and construct a falsifiable integrative model.

### 2.5. Methodological Considerations

As a non-systematic review, this study is subject to selection bias and incomplete literature retrieval; it does not constitute a systematic review and was not registered in PROSPERO. The narrative approach was chosen for the explicit goal of developing an exploratory conceptual framework rather than estimating effect sizes. Reporting transparency is provided through declaration of search dates, databases, descriptors, and selection criteria, in line with SANRA recommendations [42]. The complete search strings and full list of records screened are available from the corresponding author upon reasonable request.

## 3. Hormone-Sensitive Tissues as Neuroimmune Microenvironments

### 3.1. The Endometriotic Lesion as a Neuroimmune Microenvironment

Endometriotic lesions are now recognized as organized, dynamic microenvironments in which immune, neural, vascular, and endocrine systems interact in a self-reinforcing manner [2,23,24,25,26]. Histological and molecular evidence supports their interpretation as active algogenic niches rather than inert structural abnormalities.

Neural remodeling is a defining feature of this microenvironment. Immunohistochemistry shows markedly increased density of sensory nerve fibers, including unmyelinated C and thinly myelinated Aδ fibers [23,24,25], frequently in close proximity to ectopic glands and stroma, consistent with active neo-innervation. NGF, produced by endometrial and infiltrating immune cells [24,25,27,28], drives axonal sprouting, upregulates pronociceptive ion channels, and sensitizes primary afferents, transforming the lesion into a reorganized neural territory.

Sensory terminals show upregulation and sensitization of TRPV1 [21,22], a polymodal integrator activated by mediators such as PGE_2_, bradykinin, protons, and proinflammatory cytokines, with sustained calcium influx and lowered activation thresholds. TRPV1 expression correlates with pain severity, providing one of the most robust molecular-clinical associations in endometriosis [22].

The microenvironment also displays a distinctive immune architecture centered on mast cells. Increased densities of activated mast cells are observed in close proximity to nociceptive fibers [26]. On activation, they release histamine, tryptase, cytokines, and lipid mediators that modulate neuronal excitability, forming a local mast cell-nerve unit with bidirectional signaling [26,38,39]. In parallel, macrophage infiltration with predominantly inflammatory and profibrotic phenotypes, sustained cytokine production, and a well-characterized aromatase-dependent feedforward loop amplify local estradiol synthesis and inflammatory signaling [12,43,44]. Macrophage-derived neurotrophic and nerve-sensitizing factors, including insulin-like growth factor-1, have been identified as direct contributors to endometriosis-associated pain and may functionally complement the NGF-TRPV1 cascade [45]. Recent evidence further indicates that inflammation-mediated macrophage polarization is directly associated with TRPV1/TRPA1 heteromer expression in endometriotic lesions [46], and detailed reviews of macrophage polarization and metabolic reprogramming have refined the conceptualization of endometriotic immune microenvironments [47].

Activated afferents release CGRP and substance P, driving neurogenic inflammation (vasodilation, increased vascular permeability, immune cell recruitment) [24,37]. These processes define a coordinated neuroimmune architecture that initiates, sustains, and amplifies nociceptive signaling, providing a mechanistic substrate for the disproportion, persistence, and hormonal modulation of pain.

### 3.2. The Lipedematous Adipose Tissue as a Parallel Neuroimmune Microenvironment

Although less developed, recent evidence reveals structural and molecular features in lipedematous adipose tissue functionally consistent with those in endometriosis.

At the cellular level, lipedematous adipose tissue shows marked adipocyte hypertrophy, often independent of body mass index (BMI), with focal adipocyte necrosis and crown-like structures indicative of adipose inflammation [3,34], accompanied by increased CD68^+^ macrophage density in the dermal and subcutaneous compartments, an intrinsic feature of the phenotype [3,29].

Macrophage polarization is biologically distinctive: CD163^+^ M2-polarized macrophages predominate without proportional T lymphocyte expansion, distinguishing lipedema from secondary lymphedema [29]. The M2 secretome promotes lipid accumulation and adipogenic dysregulation [33], and may contribute to adipose reprogramming into an algogenic microenvironment.

Mast cell involvement is directly demonstrated: histological and metabolomic analyses identify mast cell infiltration with increased histamine levels that normalize under pharmacological stabilization [31], paralleling the mast cell-rich neuroimmune niches in endometriosis [26].

A second axis of convergence is local steroidogenesis and endothelial dysfunction. Increased aromatase and intracrine estradiol biosynthesis [5,30,32] indicate that lipedematous adipocytes act as active endocrine units, sustaining local hormonal signaling independently of systemic levels, while endothelial barrier dysfunction increases vascular permeability and inflammation [30]. Non-invasive sodium MRI confirms elevated tissue sodium in skin and subcutaneous adipose tissue, a quantifiable, therapy-responsive proxy of tissue inflammation [48].

Vascular remodeling reinforces this architecture, with evidence of angiogenesis, capillary dilation, perivascular fibrosis, and immune cell recruitment [3]. Interstitial fibrosis and cytokine production contribute to a fibroinflammatory phenotype that parallels structural features observed in endometriosis [2,25,34].

Functional evidence supports the presence of active nociceptive signaling. Ultrasound-guided infiltration of local anesthetic produces sustained pain reduction, indicating pharmacologically modifiable peripheral nociceptor activity [36]. Clinical features such as dermal hypersensitivity further support the presence of an active nociceptive circuit [35].

Direct characterization of NGF, TRPV1, and CGRP in lipedematous tissue remains absent. The proposed parallel is therefore a research hypothesis based on convergent upstream evidence, not a structurally established equivalence.

### 3.3. Convergence: Two Microenvironments, a Shared Architecture

When examined in parallel, both microenvironments share parenchymal expansion, macrophage infiltration, mast cell activation, endothelial dysfunction, intracrine estradiol production, angiogenesis, fibrosis, and sustained inflammatory signaling.

Recurrence of these elements across distinct tissues supports a shared neuroimmune organizational program modulated by local estrogenic signaling. Direct neural characterization, however, is established in endometriosis and absent in lipedema, defining a key boundary of this framework.

The absence of direct neural mapping does not invalidate the model: upstream mechanisms required for nociceptor sensitization are present in lipedematous tissue, and functional nociceptive activity has been documented [31,35,36]. The unresolved question is how the neural component is structurally organized within this microenvironment.

Accordingly, this framework proposes as a testable research hypothesis that a functionally relevant sensory network exists within lipedematous adipose tissue, organized in response to shared upstream signals into a structure analogous to that in endometriosis.

## 4. Shared Mechanisms of Peripheral Sensitization

The structural convergence above is pathophysiologically relevant only if shared components produce a common functional outcome, namely sustained reduction in nociceptor activation thresholds and amplified responsiveness. Within this framework, peripheral sensitization, neurogenic inflammation, and reciprocal neuroimmune signaling operate as interconnected elements of a unified amplification system.

Persistent exposure to an inflammatory microenvironment lowers nociceptor activation thresholds and enhances responsiveness to mechanical, thermal, and chemical stimuli [9,49,50], through a restricted set of inflammatory and neurotrophic signals, including prostaglandin E_2_, bradykinin, protons, tumor necrosis factor alpha (TNF-α), interleukin-1 beta (IL-1β), interleukin-6 (IL-6), and nerve growth factor, which converge on intracellular pathways regulating voltage-gated sodium channels and TRP-family receptors [9,49,50,51], shifting excitability to facilitate action potential generation and propagation.

NGF plays a central role in this cascade: it acutely modulates nociceptor excitability, drives transcriptional upregulation of pronociceptive channels, and promotes axonal sprouting [27,28,50], transforming the local network into a system with enhanced amplification capacity.

In endometriosis, elevated PGE_2_, proinflammatory cytokines, and NGF in peritoneal fluid and lesion biopsies correlate with both nerve fiber density and pain intensity [27,28,50,52]. TRPV1 upregulation and sensitization are a distal endpoint of this process and link directly to symptom severity [21,22]. Estradiol modulates this pathway via nociceptive ion channel expression [21], providing a direct interface between local steroidogenesis and peripheral sensitization.

In lipedema, the neural endpoint is not directly characterized, but upstream components of the same cascade are independently documented: consistent mast cell activation with elevated histamine [31]; increased CD163^+^ macrophages capable of producing neurotrophic factors [29,33]; local estradiol biosynthesis via CYP19A1 and hydroxysteroid 17-beta dehydrogenase 7 (HSD17B7), favoring mast cell activation and neurotrophic signaling [30,32]; and proinflammatory cytokines elevated within hypertrophic adipocytes [34].

Together these elements define a molecular environment capable of promoting nociceptor sensitization, supported clinically by dermal hypersensitivity, peripheral hyperalgesia, and responsiveness to local anesthetic infiltration [35,36].

Once sensitized, nociceptors release CGRP and substance P [9,37,50,52], which induce vasodilation, vascular permeability, immune cell recruitment, and mast cell degranulation [37,38,39,53]. Neurogenic inflammation thus amplifies the inflammatory microenvironment that initiated sensitization.

In endometriosis, CGRP-positive fibers and elevated circulating CGRP [41,52] support this mechanism directly. In lipedema, dermal erythema, pressure-induced hyperalgesia, and reactive skin responses are consistent with neurogenic inflammation [35], but direct neural mapping is unavailable.

The interaction is bidirectional: mast cell, macrophage, and stromal mediators sensitize nerve terminals while nociceptor-released neuropeptides further stimulate immune cells [38,39,53], establishing a positive-feedback loop that progressively increases system gain.

Once established, this loop sustains nociceptive output even under modest inflammatory stimuli, rendering pain primarily dependent on network amplification rather than structural lesion burden. This provides a mechanistic explanation for the dissociation between disease severity and pain intensity in both endometriosis and lipedema.

Although predominantly peripheral, these processes do not remain confined: sustained peripheral input drives central nociceptive circuits, facilitating the transition toward central amplification states, as reflected in the widespread hyperalgesia and overlap with central pain syndromes seen in a substantial proportion of patients [9,13,15,19,54].

## 5. The Mast Cell-TRPV1-CGRP Axis as a Shared Integrative Node

The mechanisms described above operate as functionally coupled components of a unified amplification system, suggesting a limited number of molecular interfaces organize the transition from local inflammation to sustained nociceptive gain.

We propose this integration as a tripartite functional axis: the mast cell as upstream effector, TRPV1 as molecular integrator of inflammatory input at primary afferents, and calcitonin gene-related peptide as downstream neuropeptide amplifier linking peripheral nociception to vascular, immune, and central consequences. This mast cell-TRPV1-CGRP axis is directly supported in endometriosis. In lipedema, it is proposed here as a research hypothesis based on convergent but indirect upstream evidence; the existence and structural organization of an analogous axis in adipose tissue remains entirely unproven and requires direct experimental validation.

Mast cells integrate hormonal, immune, and neural signals and localize close to vascular structures and nociceptive terminals [38,39]. On activation, they release preformed mediators (histamine, tryptase, TNF) followed by de novo cytokines, chemokines, eicosanoids, and neurotrophic factors including NGF [26,38], with spatial proximity to nerve terminals amplifying their impact on sensory signaling [38,39,55].

Mast cells in hormone-sensitive tissues express ERα, ERβ, and G protein-coupled estrogen receptors, which lower activation thresholds at physiological estradiol levels [38], providing a direct interface between local steroidogenesis and nociceptive amplification and a biological substrate for the hormonal dynamics observed in both diseases.

In endometriosis, mast cell infiltration is consistently demonstrated, colocalizing with sensory fibers and correlating with disease severity and pain intensity [26], and their interaction with NGF-producing elements contributes to neurotrophic remodeling and neo-innervation [26,27,28]. In lipedema, mast cell involvement is directly demonstrated by histological and metabolomic analyses showing increased mast cell density and elevated histamine that normalize under pharmacological stabilization [31]; combined with increased aromatase and intracrine estradiol production [30,32], this supports a local environment in which estrogen signaling and mast cell activation reinforce each other to sustain nociceptive signaling. This local environment is functionally consistent with, rather than equivalent to, that established in endometriosis.

Downstream of mast cell activation, inflammatory mediators converge on primary afferents, where their effects are integrated at the level of TRPV1. This receptor functions as a polymodal integrator of chemical, thermal, and inflammatory stimuli and translates the biochemical state of the microenvironment into electrical activity through membrane depolarization and calcium influx [21,22,38,40]. Experimental evidence demonstrates that histamine enhances TRPV1 responsiveness via H1 receptor activation, establishing a direct mechanistic link between mast cell activation and nociceptor sensitization [40].

In endometriosis, TRPV1 upregulation and sensitization are well established and correlate with pain intensity [21,22]. Estradiol further modulates TRPV1 expression, reinforcing the connection between hormonal signaling and nociceptive amplification. In lipedema, direct evidence of TRPV1 expression in subcutaneous adipose tissue is, to our knowledge, absent. Nevertheless, the biochemical prerequisites for TRPV1 activation, including histamine, proinflammatory cytokines, and local estradiol, are present [30,31,32,34], and the clinical phenotype is consistent with TRPV1-mediated nociception [35]. These observations support a biologically plausible role for TRPV1 signaling while defining a critical target for direct validation.

Activation of TRPV1-expressing afferents promotes the release of calcitonin gene-related peptide, a neuropeptide with established roles in vascular regulation, immune modulation, and central nociceptive processing [37]. CGRP induces vasodilation, increases vascular permeability, activates immune cells, and facilitates synaptic transmission within the dorsal horn, thereby extending the effects of peripheral nociceptive activation beyond the local tissue environment [37,53].

In endometriosis, CGRP-positive fibers are present within lesions, and circulating CGRP levels are elevated in subgroups of patients, particularly those with comorbid migraine [41,52]. In lipedema, direct evidence remains limited, but the coexistence of mast cell activation, endothelial dysfunction, and high prevalence of centrally mediated pain syndromes defines a biological context in which CGRP-mediated amplification is plausible. Accordingly, CGRP may function as a system-level amplifier linking nociceptive processes across multiple anatomical compartments, although this role remains to be directly demonstrated in lipedema.

The mast cell-nociceptor interaction is bidirectional: mast cell mediators sensitize nerve terminals, while activated nociceptors release neuropeptides that further stimulate degranulation [26,38,39]. This mast cell-nerve unit constitutes a neuroimmune microdomain in which immune and neural signaling are tightly coupled, enabling rapid amplification of nociceptive output.

Within this model, the mast cell-TRPV1-CGRP axis functions as a molecular bottleneck through which diverse upstream signals, including intracrine steroidogenesis, macrophage polarization, endothelial dysfunction, and cytokine release, are translated into a unified nociceptive output, explaining how anatomically distinct diseases generate similar pain phenotypes.

## 6. From Peripheral to Central Sensitization

The axis developed above explains peripheral amplification of nociceptive signals but does not fully account for the broader clinical phenotype of endometriosis and lipedema. In both conditions, a substantial proportion of patients exhibit features extending beyond peripheral mechanisms, including persistence of pain after anatomical control of the primary lesion, widespread hyperalgesia and allodynia, and a high prevalence of comorbid pain syndromes such as fibromyalgia, migraine, irritable bowel syndrome, painful bladder syndrome, and vulvodynia [9,13,15,19,51,54]. These patterns support central reorganization driven by sustained peripheral input.

Sustained peripheral nociceptive signaling induces activity-dependent dorsal horn synaptic changes that define central sensitization, a state of CNS hyperresponsiveness capable, in advanced stages, of sustaining pain independently of peripheral input [49]. This process involves enhanced glutamatergic transmission, upregulation and phosphorylation of NMDA and AMPA receptors, increased intracellular calcium signaling, and activation of intracellular cascades that remodel synaptic architecture [49,51,53]. These changes lower activation thresholds, expand receptive fields, and recruit previously silent synapses, resulting in amplification of nociceptive signaling and, in some cases, generation of pain in response to non-noxious stimuli.

Descending modulatory alterations further contribute: impaired inhibitory interneurons and reduced supraspinal inhibition shift the balance toward facilitation [49,53], reinforced by spinal glial activation [9,53]. These adaptations progressively decouple pain perception from the original peripheral lesion.

In endometriosis, central sensitization is supported by multiple complementary lines of evidence. Quantitative sensory testing shows generalized hyperalgesia, reduced pain thresholds, and impaired conditioned pain modulation [9,15,51,56], a pattern consistent with the nociplastic pain phenotype defined by current International Association for the Study of Pain—IASP criteria [57]. Neuroimaging identifies structural and functional alterations in regions involved in pain processing and modulation, including the thalamus, insula, and cingulate cortex, together with altered connectivity within descending modulatory networks [54,58]. Sustained peripheral input from endometriotic lesions therefore appears to drive central reorganization, which helps explain why pain often persists after surgery and why disease stage correlates weakly with symptom severity [7,9,12,15].

In lipedema, formal characterization of central sensitization remains limited, but convergent clinical and epidemiological observations support its presence in a substantial subgroup of patients. The high prevalence of fibromyalgia, observed in approximately 35 percent of patients, provides a clinically meaningful proxy of centrally amplified pain states [19]. Additional observations indicate that many patients exhibit pre-existing psychological vulnerability, including anxiety, depression, and post-traumatic stress disorder, which are associated with increased pain intensity and may contribute to amplification of nociceptive processing [13]. These findings are consistent with epidemiological evidence demonstrating a non-random co-occurrence of lipedema with gynecologic and pain-related conditions across cohorts [14], supporting the interpretation that central vulnerability may precede and subsequently amplify the impact of peripheral disease, although direct neurophysiological validation remains limited.

A complementary perspective comes from the interaction between pain, physical inactivity, and psychological distress: non-linear relationships indicate that high pain combined with reduced activity produces disproportionate deterioration of physical and mental health, consistent with centrally amplified states [20]. These observations support a contribution of central sensitization to the lipedema phenotype while underscoring the need for direct experimental confirmation.

A particularly informative observation provides mechanistic insight into the role of local steroid metabolism in pain modulation. A missense variant in the AKR1C1 gene, involved in progesterone and neurosteroid metabolism, has been identified in familial lipedema [59]. Reduced enzymatic activity leads to accumulation of allopregnanolone, a potent positive modulator of gamma-aminobutyric acid type A receptor (GABA_A) receptors with established analgesic properties. Affected individuals exhibit typical structural features of lipedema but absence of pain, providing a rare example of dissociation between tissue phenotype and nociceptive output. As this finding derives from a single family [59], it is informative but requires independent replication and should not be interpreted as sufficient to explain the broader lipedema phenotype.

Central sensitization also enables integration of nociceptive signals across anatomical territories. Visceral and somatic afferents converge onto shared dorsal horn neurons, and under sensitized conditions, reduced inhibition and expanded receptive fields permit cross-organ interactions [9,15,60]. This mechanism provides a biological basis for the overlap between endometriosis and other pain syndromes and is consistent with similar patterns observed in lipedema [9,13,15,19,20]. Once central sensitization is established, nociceptive input from multiple hormone-sensitive tissues may be integrated within a shared amplified network, offering one plausible framework for interpreting the epidemiological coexistence of both conditions [14].

Together, these mechanisms extend the framework beyond local tissue processes into a system-level model but do not explain the temporal clustering of symptoms within reproductive transitions, addressed next as hormonal chronodependence.

## 7. Hormonal Chronodependence

The mechanisms developed above explain how the algogenic state is sustained, but not when it is initiated or why disease course is coupled to reproductive transitions. This temporal dimension defines periods of vulnerability during which the underlying neuroimmune system becomes clinically manifest or destabilized.

Both diseases cluster onset and exacerbation at puberty, pregnancy, and menopause. In endometriosis, onset frequently coincides with or follows menarche, with activity modulated by cyclical hormonal changes across reproductive life [1,7]. Suppression of ovarian function yields only partial improvement; pain often persists with established central sensitization, and the effect of menopause is variable [7,12].

Lipedema shows a similar pattern: onset or worsening reported in 62–72% at puberty, 53% during pregnancy, and 67.9% at menopause [14], paralleling endometriosis and consistent with shared tissue-level hormonal vulnerability; as these proportions originate from a single cohort dataset, they require independent replication.

A critical divergence emerges at menopause. Endometriosis often improves with declining ovarian estradiol, whereas lipedema frequently worsens or arises de novo during this transition [14]. This paradox indicates that hormonal dependence in lipedema cannot be explained by circulating estrogen levels alone, and points to tissue-specific mechanisms capable of sustaining or amplifying local signaling under systemic hormonal decline. This observation is mechanistically informative, as it suggests a shift from ovarian to peripheral steroidogenesis and highlights the potential roles of local estradiol production, altered steroid metabolism, and reduction of progesterone-derived neurosteroids as drivers of disease destabilization [6,14,59].

The molecular basis is intracrine steroidogenesis: unlike systemic endocrine signaling, intracrine mechanisms allow tissues to regulate their hormonal environment independently of circulating levels, maintaining active estrogen signaling even under systemic suppression.

In endometriosis, ectopic lesions show aberrant aromatase expression and a PGE_2_-aromatase-estradiol-COX-2 feedforward loop sustaining local estradiol production [12,21]. Dysregulation of 17β-HSD enzymes further accumulates active estradiol.

In lipedema, increased aromatase [30] and upregulated HSD17B7, LIPE, and steroid sulfatase [5,32] in adipocytes support tissue-selective estradiol biosynthesis. The parallel is mechanistically consistent but not directly equivalent to endometriosis.

Tissues sustaining intracrine estrogen production are inherently less responsive to systemic hormonal modulation, which plausibly explains incomplete therapeutic responses and persistence of symptoms during apparent hormonal stability.

Reproductive transitions involve rapid systemic hormonal fluctuation requiring intracrine adaptation; they may thus act as destabilizing events that trigger onset or exacerbation through transient imbalance between systemic and local regulation.

The AKR1C1 pathway provides an additional layer of regulation linking steroid metabolism to nociceptive modulation. This enzyme regulates both progesterone metabolism and the inactivation of allopregnanolone, a neurosteroid with inhibitory effects on nociceptive circuits [59]. During menopause, reduction of progesterone and its neuroactive derivatives may shift the balance toward enhanced nociceptive amplification. Variability in AKR1C1 activity may therefore influence both structural and pain phenotypes, providing a biologically plausible explanation for the worsening of lipedema under conditions of declining systemic estrogen levels [6,14,59]. This observation is informative but should not be interpreted as sufficient to explain the broader disease phenotype.

Together, these observations support a shared biological architecture centered on intracrine steroidogenesis. The temporal pattern is a reproducible population-level signal consistent with the proposed framework, though not by itself sufficient to establish it.

## 8. Pain Flares as Events of Neuroimmune Network Instability

The preceding sections describe a nociceptive system reorganized into a sensitized, estrogen-responsive, dynamically modulated state. Episodic exacerbations are a prominent but insufficiently explained feature of both conditions, frequently arising unpredictably without measurable lesional progression or overt hormonal destabilization [7,9,12,13], and within a broader clinical context in which lipedema is consistently associated with gynecologic and pain-related symptom clusters across cohorts [14].

**Figure 3:**
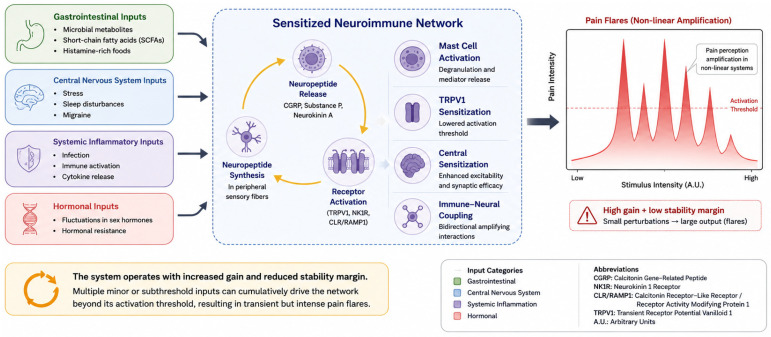
Pain flares as neuroimmune network instability events. Multiple gastrointestinal, central nervous system, systemic inflammatory, and hormonal inputs converge on a sensitized neuroimmune network characterized by mast cell activation, TRPV1 sensitization, neuropeptide signaling, immune–neural coupling, and central sensitization. Within this high-gain state, reciprocal interactions between peripheral and central components may amplify nociceptive processing and reduce network stability. As a result, minor or subthreshold perturbations can cumulatively drive the system beyond its activation threshold, generating disproportionately amplified pain responses and transient pain flares. The circular pathway represents a proposed amplifying feedback mechanism involving neuropeptide synthesis, receptor activation, and neuropeptide release. The model is conceptual and intended to illustrate network dynamics rather than a validated mechanistic sequence. Abbreviations: TRPV1, transient receptor potential vanilloid 1; CGRP, calcitonin gene-related peptide; NK1R, neurokinin-1 receptor; CLR/RAMP1, calcitonin receptor-like receptor/receptor activity-modifying protein 1.

We interpret these events as transient instabilities of a sensitized neuroimmune network operating near its activation threshold (Figure 3), reflecting an underlying multisystem phenotype rather than isolated tissue events.

Contemporary neurobiology conceptualizes chronic pain as a disorder of network dynamics [49,53]. In this state the nociceptive system shows increased gain, reduced activation thresholds, and non-linear behavior, such that minor input fluctuations can generate disproportionately amplified responses [12,49].

Within this framework, flares are not discrete increases in inflammatory burden but transient excursions of a sensitized system beyond its stability margin, with magnitude depending less on triggering intensity than on the instantaneous gain of the network and its residual regulatory capacity [12,38,49]. This accounts for unpredictability, disproportion to triggers, temporal clustering, and persistence despite apparent disease stability [7,9,12,13,15,19].

Multiple mechanisms reduce this stability margin: bidirectional mast cell-nerve coupling forms a local amplification loop in which small perturbations produce large excitability changes [26,38,39]; central sensitization reduces descending inhibitory control [49,53]; and cross-organ sensitization expands the inputs that can activate the system [9,15].

A key consequence is that the anatomical site of pain need not correspond to the origin of the triggering event. Flares preferentially appear in territories with the lowest activation threshold, reflecting nociceptive memory rather than local pathology [12,49]; examples include pelvic pain triggered by gastrointestinal perturbations, limb pain by psychological stress, or cephalic pain by systemic inflammatory inputs [13,16,19,41].

If flares reflect network instability rather than isolated lesional activity, their classification could plausibly be based on the system that delivers the destabilizing input rather than on the anatomical location of pain. Accordingly, Table 1 summarizes the proposed four-class phenotyping model, its principal triggers, putative mechanisms, and conceptual research targets. As a research proposal, not a clinically validated taxonomy, we outline below a four-class phenotyping model intended to guide mechanistic investigation and exploratory patient stratification. The model has not been prospectively tested and should not be used for clinical classification.

These phenotypes are not mutually exclusive and may coexist within an individual, their relative contribution varying across the reproductive lifespan [12,13,15,38]. Longitudinal characterization of flare directionality may identify dominant vulnerabilities and inform targeted research, consistent with the heterogeneous but recurrent clusters observed in lipedema [14].

The histamine-dominant category is particularly relevant in lipedema. Microbiota-derived lipopolysaccharide may accumulate in hormonally responsive adipose depots, activating TLR4 and NLRP3 inflammasome signaling and sustaining chronic inflammation [61]; a parallel gut-pelvis interaction is recognized in endometriosis through the estrobolome, in which microbial β-glucuronidase activity modulates estrogen load [46]. In tissues primed by mast cell activation and low-grade endotoxemia, acute gut-derived perturbations could plausibly amplify inflammatory and neuroimmune pathways. This is a research hypothesis based on convergent but indirect data; the gastrointestinal trigger pathway for lipedema flares has not been directly demonstrated.

This model links gastrointestinal perturbations to pain flares in lipedema and extends mast cell-nerve concepts [31,38,39]. A comparable, less well-defined gut-pelvis interaction is described in endometriosis [9,15], suggesting a testable framework for both conditions.

Reinterpreting pain flares as manifestations of neuroimmune network instability integrates peripheral, central, and systemic contributors into a unified model of symptom dynamics, in which flares express a broader, non-random clustering of hormone-sensitive and pain-related conditions across populations [14]. This transforms flares from unpredictable events into mechanistically tractable phenomena and provides a foundation for targeted investigation of their triggers and modulation.

## 9. Translational Implications and a Shared Research Agenda

The translational value of this framework lies in generating testable predictions. Epidemiological evidence that lipedema clusters with hormone-sensitive gynecologic and pain-related conditions across populations [14] supports coordinated investigation across these traditionally separate domains.

This framework produces accessible experimental predictions. Immunohistochemistry standardized in endometriosis, when applied to lipedematous adipose tissue, would be predicted to reveal increased sensory nerve density, NGF expression, TRPV1 sensitization, and CGRP- or substance P-positive fibers in proximity to mast cells [21,22,23,24,25,26,27,28,52]. Such findings would provide direct validation of the proposed axis.

It further predicts measurable central sensitization in lipedema by QST and functional neuroimaging, particularly in patients with onset during hormonally dynamic periods [9,51,54,58]; direct comparison with endometriosis cohorts would test this.

Mast cell stabilization may reduce pain intensity and flare frequency in both conditions if the mechanism is confirmed. Preliminary support exists in lipedema [31], whereas systematic evaluation in endometriosis remains limited despite a strong rationale [26]. Modulation of peripheral nociceptor activity [36] and of CGRP signaling, validated in migraine through anti-CGRP and anti-receptor monoclonal antibodies [16,41,62], are plausible research targets at distinct nodes of the proposed axis, with no established clinical role in endometriosis or lipedema. The framework also predicts that patients with coexistent disease may show greater nociceptive amplification than either alone [14].

The framework supports mechanism-based over anatomy-based stratification, since traditional staging correlates poorly with symptoms in both diseases [3,4,7,8,9,11]. A minimal stratification dataset might include QST for peripheral and central sensitization, longitudinal flare characterization, and biomarker profiling of mast cell mediators, CGRP, and endotoxemia [31,41,61], potentially identifying clinically meaningful subgroups.

**Figure 4:**
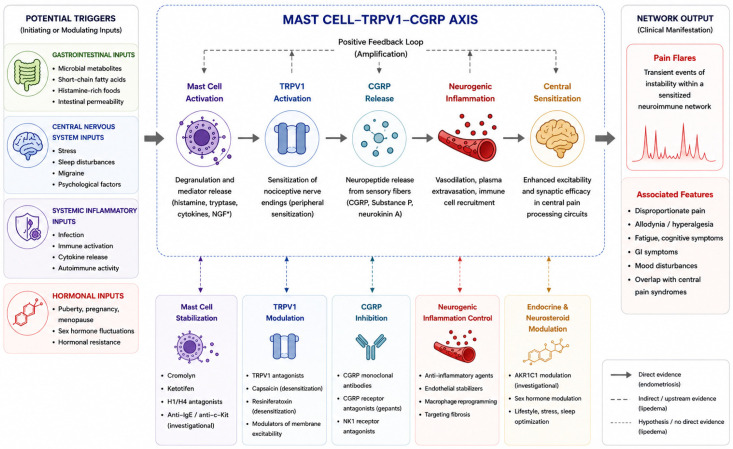
Proposed research framework highlighting potential intervention points along the mast cell–TRPV1–CGRP axis. The proposed neuroimmune framework conceptualizes pain amplification as a dynamic process linking mast cell activation, TRPV1 sensitization, CGRP-mediated neurogenic inflammation, and central sensitization. Potential gastrointestinal, central nervous system, systemic inflammatory, and hormonal inputs may converge on this sensitized network, generating pain flares and persistent nociceptive amplification. The lower panel illustrates candidate intervention points positioned along different levels of the proposed pathway, including mast cell stabilization, TRPV1 modulation, CGRP pathway inhibition, modulation of neurogenic inflammation, and endocrine or neurosteroid-related mechanisms. These intervention points are presented exclusively as hypothesis-generating research targets and do not represent established therapeutic strategies for either endometriosis or lipedema. Solid arrows indicate mechanisms directly supported by evidence in endometriosis. Dashed arrows indicate indirect or upstream evidence currently available in lipedema. Dotted arrows represent hypothetical mechanisms requiring direct experimental validation. Abbreviations: TRPV1, transient receptor potential vanilloid 1; CGRP, calcitonin gene-related peptide; NGF, nerve growth factor; AKR1C1, aldo-keto reductase family 1 member C1.

The convergence of both diseases on a shared neuroimmune axis further suggests that multilevel therapeutic targeting may be required to effectively modulate nociceptive amplification within this system (Figure 4).

Pharmacological modulation of intracrine steroidogenesis, including progestogens with anti-estrogenic activity, may be a plausible research target for reducing estrogen-driven amplification [14]. Mast cell stabilization [31], peripheral nociceptor modulation [36,40], CGRP modulation [16,41], and the AKR1C1-allopregnanolone axis [59] each merit mechanistic investigation as candidate targets; none currently constitutes an evidence-based clinical recommendation for either condition.

The multi-level organization of the proposed axis suggests that combined approaches addressing multiple nodes may be required to meaningfully modify nociceptive dynamics; this prediction itself remains entirely to be tested.

Research priorities follow: direct characterization of neural and mast cell components in lipedematous tissue; formal assessment of central sensitization in lipedema; systematic mast cell biology across both conditions; prospective studies with structured flare phenotyping; exploratory parallel investigations of mast cell stabilization, CGRP modulation, and peripheral nociceptor activity; and characterization of intracrine steroidogenic pathways.

Coordinated pursuit of these priorities would move the framework from conceptual synthesis to empirical evaluation.

## 10. Alternative Interpretations of Epidemiological Convergence

The epidemiological parallels between endometriosis and lipedema, including their female predominance, symptom clustering across reproductive transitions, and frequent co-occurrence with centrally mediated pain syndromes [13,14,19], are consistent with the proposed neuroimmune framework but do not by themselves establish it. Several alternative interpretations could contribute to the observed convergence and are considered here to avoid overinterpretation of correlational data.

First, both conditions affect women and are modulated by puberty, pregnancy, and menopause. This shared temporal behavior may reflect common exposure to systemic hormonal fluctuation acting independently on two hormone-sensitive tissues, rather than a shared neuroimmune mechanism. Parallel hormonal responsiveness is therefore compatible with, but not specific to, the proposed axis.

Second, the apparent co-occurrence may be partly attributable to ascertainment. Women with one chronic female-predominant condition typically undergo more medical consultations, investigations, and diagnoses, which can inflate the measured coexistence of two such conditions through ascertainment and referral bias. Reported clustering may thus overestimate any true biological association.

Third, the frequent overlap with fibromyalgia, migraine, and irritable bowel syndrome [9,13,15,19] may reflect a shared predisposition to central sensitization or a broader nociplastic pain phenotype, rather than a common peripheral mast cell-TRPV1-CGRP axis. Under this interpretation, the convergence would arise at the level of central pain processing and would not require an analogous peripheral mechanism in both tissues.

Although these explanations may each contribute to the observed epidemiological overlap, they do not fully account for the convergence of inflammatory, endocrine, vascular, and neuroimmune findings summarized in the present framework and in Table 2. The proposed model should therefore be regarded as one biologically plausible interpretation among several, to be discriminated from these alternatives by the direct experimental tests outlined above.

## 11. Limitations of the Proposed Framework

This framework rests on a convergent but incomplete body of evidence and extrapolates from a well-characterized condition (endometriosis) to a comparatively underexplored one (lipedema). Each limitation below corresponds to a testable component and a research priority.

The principal limitation is asymmetric direct neural characterization. In endometriosis, NGF, TRPV1 sensitization, CGRP-positive fibers, and mast cell-nerve interactions are supported by molecular and histological evidence [21,22,23,24,25,26,27,28,52]. In lipedema, equivalent characterization is limited and support is indirect, resting on upstream sensitizing mediators [3,29,30,31,32,33], clinical features consistent with neurogenic inflammation [35], and responsiveness to local anesthetic infiltration [36], none of which replace direct validation, the primary research priority.

A second limitation is the absence of studies directly comparing both diseases under unified methodologies; the framework integrates heterogeneous designs and populations, so some apparent convergence may reflect methodological overlap rather than biological identity. Prospective studies applying standardized histology, molecular profiling, neurophysiology, and imaging across both conditions are needed to define the limits of the proposed overlap.

The flare phenotyping framework also requires validation: it is conceptually consistent with available clinical observations [13,14,20,31] but has not been evaluated in prospective cohorts. The proposed role of gut-derived endotoxemia [61] is also hypothetical.

Biological heterogeneity is a further limitation: endometriosis includes multiple phenotypes [1,7,9], and lipedema varies in distribution, stage, and remodeling [3,4,5,6,11]. The framework applies most directly to patients with persistent, disproportionate, hormonally modulated pain, particularly with central sensitization and comorbid pain syndromes, and less where structural, metabolic, or mechanical factors predominate.

Finally, the therapeutic implications are hypothesis-generating rather than evidence-based. With the partial exception of established hormonal therapies in endometriosis, interventions targeting mast cells, CGRP signaling, peripheral nociceptors, or neurosteroid pathways lack sufficient clinical evidence for routine use and require controlled studies designed to test the proposed mechanisms.

Together, these limitations define the current boundaries of the model while delineating a structured research agenda, reinforcing its role as a research-stage synthesis that integrates existing evidence and directs future investigation toward testable questions.

## 12. Conclusions

Endometriosis and lipedema are chronic, female-predominant, hormone-sensitive disorders whose most disabling feature, persistent pain disproportionate to anatomical lesion burden, remains insufficiently explained by tissue-centered models. In both, pain persists despite anatomical control of the lesion [3,7,8,9,36], exacerbations occur under stable hormonal conditions [12,13], comorbid pain syndromes are prevalent [13,15,16,19,20], and onset clusters within reproductive transitions [1,14].

These parallels have not been previously integrated into a unified mechanistic framework.

We propose that endometriosis and lipedema may be conceptualized as topographically distinct expressions of a shared neuroimmune axis in hormone-sensitive tissues, in which steroid-responsive tissues are functionally reprogrammed under local estrogenic modulation into algogenic microenvironments converging on the mast cell-TRPV1-CGRP integrative pathway.

Convergence of structural, molecular, and clinical features across both conditions supports the plausibility of this model. Its evidentiary basis remains asymmetric, with direct mechanistic support in endometriosis and convergent but indirect evidence in lipedema, which defines current limits and validation priorities.

Two observations provide preliminary support for this model. First, mast cell infiltration and elevated histamine levels in lipedematous adipose tissue, together with their reversibility under pharmacological stabilization [31], establish a functional parallel with endometriosis at the level of upstream neuroimmune signaling. Second, the identification of a loss-of-function AKR1C1 variant associated with absence of pain in a single family with otherwise typical lipedema [59] suggests a potential link between intracrine steroid metabolism and nociceptive modulation, highlighting a possible endocrine–neural regulatory interface that requires independent replication.

Beyond mechanistic interpretation, this unification suggests endometriosis and lipedema may be more productively studied as related manifestations of a shared neuroimmune process, supporting coordinated investigation across gynecology, endocrinology, and pain biology. The derived research agenda, comprising validation of the proposed axis, mechanism-based stratification, and targeted therapeutic exploration, provides a structured pathway for future studies.

Reframing these diseases within a shared neuroimmune context does not diminish the importance of their anatomical substrates. It expands the interpretive model to incorporate dynamic interactions between local tissue biology and system-level nociceptive regulation.

If validated, this framework supports the view that persistent pain in these disorders may be more appropriately understood as a form of neuroimmune network pathology.

## Figures and Tables

**Table 1:** Proposed neuroimmune flare phenotypes in endometriosis and lipedema (research framework, not a clinical classification).

Flare Phenotype	Primary Entry System	Typical Clinical Triggers	Predominant Direction of Propagation	Dominant Pathophysiological Mechanisms	Conceptual Research Targets
Histamine-dominant/gut-related	Gastrointestinal tract	Histamine-rich foods; ultra-processed diet; intestinal distension; constipation; microbiota fluctuations; low-grade endotoxemia	Gut → pelvis/limbs ± headache	Mast cell activation; histamine release; visceral afferent sensitization; TRPV1 activation; LPS-TLR4-NLRP3 signaling	Mast cell stabilization; reduction of histaminergic load; intestinal barrier modulation
Central-dominant (sleep/stress/migraine)	Central nervous system (CNS)	Sleep deprivation; psychological stress; migraine; fatigue; post-traumatic stress disorder (PTSD)	Head → pelvis/limbs	Increased central gain; impaired descending inhibition; trigeminovascular activation; CGRP signaling; stress-induced hyperalgesia	CGRP modulation; restoration of inhibitory control; sleep regulation; psychological intervention
Systemic inflammatory	Systemic immune system	Infections; allergic reactions; systemic inflammation	Systemic → pelvis/limbs ± headache	Systemic mast cell activation; cytokine spill-over; neurogenic inflammation; global nociceptive facilitation	Control of systemic inflammation; mast cell modulation
Residual hormonal	Peripheral neuroendocrine axis	Residual estradiol fluctuations; incomplete suppression; perimenopause	Variable	Estradiol-mediated facilitation; TRPV1 sensitization; mast cell activation; instability of intracrine steroidogenesis	Hormonal stabilization; modulation of intracrine pathways

Legends: TRPV1, transient receptor potential vanilloid 1; CGRP, calcitonin gene-related peptide; CNS, central nervous system; PTSD, post-traumatic stress disorder; LPS, lipopolysaccharide; TLR4, Toll-like receptor 4; NLRP3, NOD-like receptor family pyrin domain containing 3.

**Table 2:** Convergent and divergent neuroimmune features in endometriosis and lipedema.

Domain/Mechanism	Endometriosis (Direct Evidence)	Lipedema (Current Evidence)	Evidence Asymmetry
Sensory nerve fiber density	Increased C and Aδ fiber density in lesions; immunohistochemistry confirmed [23,24,25,52]	Not directly characterized in subcutaneous adipose tissue; clinical hyperalgesia and dermal hypersensitivity present [35,36]	Direct in endometriosis; inferred in lipedema (priority for validation)
Nerve growth factor (NGF)	Elevated expression in lesions and peritoneal fluid; correlates with pain intensity [24,25,27,28]	Not directly demonstrated; M2 macrophages capable of NGF production are present [29,33]	Direct in endometriosis; mechanistically plausible in lipedema
TRPV1 sensitization	Upregulation correlated with pain severity; estradiol-modulated [21,22]	Not directly demonstrated; upstream prerequisites (histamine, cytokines, estradiol) present [30,31,32,34]	Direct in endometriosis; functionally plausible in lipedema
CGRP/neurogenic inflammation	CGRP-positive fibers in lesions; elevated circulating CGRP in migraine-comorbid subgroups [41,52]	Indirect: dermal erythema, pressure-induced hyperalgesia, neurogenic-pattern clinical signs [35]	Direct in endometriosis; clinical-phenotype-level evidence in lipedema
Mast cell infiltration	Increased density colocalized with nerve fibers; correlates with pain [26]	Directly demonstrated; elevated histamine reversible under cromolyn [31]	Symmetrical—directly demonstrated in both
Macrophage polarization	Predominantly inflammatory and profibrotic phenotypes [2,12]	CD163+ M2-polarized macrophages predominate [29,33]	Direct in both; phenotype differs (M1-leaning vs M2-leaning)
Intracrine estrogen biosynthesis	Aromatase-driven local estradiol; feedforward PGE2-aromatase loop [12,21]	Increased aromatase, HSD17B7, LIPE, steroid sulfatase in adipocytes [5,30,32]	Symmetrical—directly demonstrated in both
Endothelial dysfunction/angiogenesis	Neoangiogenesis, perivascular fibrosis, increased vascular permeability [2]	Capillary dilation, perivascular fibrosis, increased endothelial permeability [3,30]	Symmetrical—directly demonstrated in both
Central sensitization	QST hyperalgesia, neuroimaging alterations, impaired conditioned pain modulation [9,15,51,54,58]	Indirect: 35% fibromyalgia prevalence; comorbid affective disorders [13,19,20]	Direct in endometriosis; proxy-level evidence in lipedema
Hormonal chronodependence	Onset/exacerbation at menarche, pregnancy, perimenopause; improves with menopause in most cases [1,7]	Onset/exacerbation at puberty (62–72%), pregnancy (53%), menopause (67.9%); often worsens at menopause [14]	Symmetrical pattern but divergent behavior at menopause
Neurosteroid regulation (AKR1C1)	Not specifically characterized as disease driver	AKR1C1 loss-of-function variant associated with absence of pain in a single family with lipedema; requires replication [59]	Specific to lipedema—provides genetic dissociation evidence
Gut-derived endotoxemia (LPS-TLR4-NLRP3)	Gut-pelvis interactions described; mechanistic detail limited [9,15]	Postulated as driver of histamine-dominant flares [61]	Hypothesized in both; mechanistically more developed in lipedema
Pharmacological reversibility (proof-of-concept)	Lesion resection partial; hormonal suppression partial [7,8,9,12]	Local anesthetic infiltration reduces pain; sodium cromoglycate normalizes histamine [31,36]	Both partial; mechanism-targeted interventions show signal in lipedema

Note: numbers in brackets refer to references listed at the end of the manuscript. “Direct evidence” indicates immunohistochemical, molecular, electrophysiological, or imaging demonstration in human tissue; “indirect/inferred” indicates clinical-phenotypic or upstream-mechanism support.

## Data Availability

No new data were created or analyzed in this study. The authors affirm that this manuscript provides an accurate and transparent account of the study. The narrative, non-systematic nature of the review and its limitations are clearly acknowledged.

## Abbreviations

AKR1C1	aldo-keto reductase family 1 member C1
AMPA	α-amino-3-hydroxy-5-methyl-4-isoxazolepropionic acid receptor
ASRM	American Society for Reproductive Medicine
BMI	Body Mass Index
CD163	Cluster of Differentiation 163
CD68	Cluster of Differentiation 68
CGRP	calcitonin gene-related peptide
CNS	central nervous system
COX-2	cyclooxygenase-2
CYP19A1	Cytochrome P450 Family 19 Subfamily A Member 1 (aromatase gene)
ERα	estrogen receptor alpha
ERβ	estrogen receptor beta
GABA	gamma-aminobutyric acid
HSD17B7	hydroxysteroid 17-beta dehydrogenase 7
IASP	International Association for the Study of Pain
IBS	Irritable Bowel Syndrome
IL-1β	interleukin-1 beta
IL-6	interleukin-6
LPS	lipopolysaccharide
M1	Macrophage Phenotype 1
M2	Macrophage Phenotype 2
MeSH	Medical Subject Headings
MRI	Magnetic Resonance Imaging
NGF	nerve growth factor
NLRP3	NOD-like receptor family pyrin domain containing 3
NMDA	N-methyl-D-aspartate
PGE_2_	Prostaglandin E_2_
PTSD	post-traumatic stress disorder
QST	Quantitative Sensory Testing
TLR4	Toll-like receptor 4
TNF-α	tumor necrosis factor alpha
TRPA1	Transient Receptor Potential Ankyrin 1
TRPV1	Transient Receptor Potential Vanilloid 1

## References

[1] Zondervan K.T., Becker C.M., Koga K., Missmer S.A., Taylor R.N., Viganò P. (2018). Endometriosis. Nat. Rev. Dis. Primers.

[2] Asante A., Taylor R.N. (2011). Endometriosis: The Role of Neuroangiogenesis. Annu. Rev. Physiol..

[3] AL-Ghadban S., Cromer W., Allen M., Ussery C., Badowski M., Harris D., Herbst K.L. (2019). Dilated Blood and Lymphatic Microvessels, Angiogenesis, Increased Macrophages, and Adipocyte Hypertrophy in Lipedema Thigh Skin and Fat Tissue. J. Obes..

[4] Mortada H., Alhithlool A.W., AlBattal N.Z., Shetty R.K., AL-Mekhlafi G.A., Hong J.P., Alshomer F. (2025). Lipedema: Clinical Features, Diagnosis, and Management. Arch. Plast. Surg..

[5] Poojari A., Dev K., Rabiee A. (2022). Lipedema: Insights into Morphology, Pathophysiology, and Challenges. Biomedicines.

[6] Katzer K., Hill J.L., McIver K.B., Foster M.T. (2021). Lipedema and the Potential Role of Estrogen in Excessive Adipose Tissue Accumulation. Int. J. Mol. Sci..

[7] Stratton P., Berkley K.J. (2011). Chronic Pelvic Pain and Endometriosis: Translational Evidence of the Relationship and Implications. Hum. Reprod. Update.

[8] Vercellini P., Fedele L., Aimi G., Pietropaolo G., Consonni D., Crosignani P.G. (2007). Association between Endometriosis Stage, Lesion Type, Patient Characteristics and Severity of Pelvic Pain Symptoms: A Multivariate Analysis of over 1000 Patients. Hum. Reprod..

[9] Maddern J., Grundy L., Castro J., Brierley S.M. (2020). Pain in Endometriosis. Front. Cell. Neurosci..

[10] Biasioli A., Previtera F., Mazzera I., Degano M., Zermano S., Tius V., Piacenti I., Seracchioli R., Raimondo D., Martina M.D. (2026). Central Sensitization in Women with Endometriosis: A Cross-Sectional Study. BMC Women’s Health.

[11] Kamamoto F., Baiocchi J.M.T., Batista B.N., Ribeiro R.D.A., Modena D.A.O., Gornati V.C. (2024). Lipedema: Exploring Pathophysiology and Treatment Strategies—State of the Art. J. Vasc. Bras..

[12] Brawn J., Morotti M., Zondervan K.T., Becker C.M., Vincent K. (2014). Central Changes Associated with Chronic Pelvic Pain and Endometriosis. Hum. Reprod. Update.

[13] Erbacher G., Bertsch T. (2020). Lipoedema and Pain: What is the role of the psyche?—Results of a pilot study with 150 patients with Lipoedema. Phlebologie.

[14] Viana D.P.D.C., Invitti A.L., Schor E. (2026). Lipedema in Women and Its Interrelationship with Endometriosis and Other Gynecologic Diseases: A Scoping Review. Biomedicines.

[15] McNamara H.C., Frawley H.C., Donoghue J.F., Readman E., Healey M., Ellett L., Reddington C., Hicks L.J., Harlow K., Rogers P.A.W. (2021). Peripheral, Central, and Cross Sensitization in Endometriosis-Associated Pain and Comorbid Pain Syndromes. Front. Reprod. Health.

[16] Colombo G.E., Makieva S., Somigliana E., Schoretsanitis G., Leeners B., Polli C., Salmeri N., Kalaitzopoulos D.R., Vigano’ P. (2025). The Association between Endometriosis and Migraine: A Systematic Review and Meta-Analysis of Observational Studies. J. Headache Pain.

[17] Berkley K. (2005). A Life of Pelvic Pain. Physiol. Behav..

[18] Wesselmann U. (2001). Neurogenic Inflammation and Chronic Pelvic Pain. World J. Urol..

[19] Cagliyan Turk A., Erden E., Eker Buyuksireci D., Umaroglu M., Borman P. (2024). Prevalence of Fibromyalgia Syndrome in Women with Lipedema and Its Effect on Anxiety, Depression, and Quality of Life. Lymphat. Res. Biol..

[20] Aitzetmüller-Klietz M.-L., Busch L., Hamatschek M., Paul M., Schriek C., Wiebringhaus P., Aitzetmüller-Klietz M., Kückelhaus M., Hirsch T. (2023). Understanding the Vicious Circle of Pain, Physical Activity, and Mental Health in Lipedema Patients: A Response Surface Analysis. J. Clin. Med..

[21] Greaves E., Grieve K., Horne A.W., Saunders P.T.K. (2014). Elevated Peritoneal Expression and Estrogen Regulation of Nociceptive Ion Channels in Endometriosis. J. Clin. Endocrinol. Metab..

[22] Rocha M.G., e Silva J.C.R., Ribeiro Da Silva A., Candido Dos Reis F.J., Nogueira A.A., Poli-Neto O.B. (2011). TRPV1 Expression on Peritoneal Endometriosis Foci Is Associated with Chronic Pelvic Pain. Reprod. Sci..

[23] Anaf V. (2000). Relationship between Endometriotic Foci and Nerves in Rectovaginal Endometriotic Nodules. Hum. Reprod..

[24] Tokushige N., Markham R., Russell P., Fraser I.S. (2006). High Density of Small Nerve Fibres in the Functional Layer of the Endometrium in Women with Endometriosis. Hum. Reprod..

[25] Mechsner S., Schwarz J., Thode J., Loddenkemper C., Salomon D.S., Ebert A.D. (2007). Growth-Associated Protein 43–Positive Sensory Nerve Fibers Accompanied by Immature Vessels Are Located in or near Peritoneal Endometriotic Lesions. Fertil. Steril..

[26] Anaf V., Chapron C., El Nakadi I., De Moor V., Simonart T., Noël J.-C. (2006). Pain, Mast Cells, and Nerves in Peritoneal, Ovarian, and Deep Infiltrating Endometriosis. Fertil. Steril..

[27] Kajitani T., Maruyama T., Asada H., Uchida H., Oda H., Uchida S., Miyazaki K., Arase T., Ono M., Yoshimura Y. (2013). Possible Involvement of Nerve Growth Factor in Dysmenorrhea and Dyspareunia Associated with Endometriosis. Endocr. J..

[28] Barcena De Arellano M.L., Arnold J., Lang H., Vercellino G.F., Chiantera V., Schneider A., Mechsner S. (2013). Evidence of Neurotrophic Events Due to Peritoneal Endometriotic Lesions. Cytokine.

[29] Felmerer G., Stylianaki A., Hollmén M., Ströbel P., Stepniewski A., Wang A., Frueh F.S., Kim B.-S., Giovanoli P., Lindenblatt N. (2020). Increased Levels of VEGF-C and Macrophage Infiltration in Lipedema Patients without Changes in Lymphatic Vascular Morphology. Sci. Rep..

[30] Strohmeier K., Hofmann M., Jacak J., Narzt M.-S., Wahlmueller M., Mairhofer M., Schaedl B., Holnthoner W., Barsch M., Sandhofer M. (2022). Multi-Level Analysis of Adipose Tissue Reveals the Relevance of Perivascular Subpopulations and an Increased Endothelial Permeability in Early-Stage Lipedema. Biomedicines.

[31] Bonetti G., Michelini S., Donato K. (2023). Targeting Mast Cells: Sodium Cromoglycate as a Possible Treatment of Lipedema. Clin. Ter..

[32] Al-Ghadban S., Isern S.U., Herbst K.L., Bunnell B.A. (2024). The Expression of Adipogenic Marker Is Significantly Increased in Estrogen-Treated Lipedema Adipocytes Differentiated from Adipose Stem Cells In Vitro. Biomedicines.

[33] Grewal T., Kempa S., Buechler C. (2025). Lipedema: A Disease Triggered by M2 Polarized Macrophages?. Biomedicines.

[34] Schimak E., Steiner M., Lipp A.-T., Bauer H.-C., Bauer H., Ernst A.M. (2024). Lipedema Adipocytes in Culture: Signs of Hypertrophy, Inflammation, and Fibrosis. Adipose Tissue—Development, Homeostasis, and Remodelling.

[35] Chakraborty A., Crescenzi R., Usman T.A., Reyna A.J., Garza M.E., Al-Ghadban S., Herbst K.L., Donahue P.M.C., Rutkowski J.M. (2022). Indications of Peripheral Pain, Dermal Hypersensitivity, and Neurogenic Inflammation in Patients with Lipedema. Int. J. Mol. Sci..

[36] Mendoza E. (2026). A New Approach to Pain Management in Lipedema. Vasa.

[37] Russell F.A., King R., Smillie S.-J., Kodji X., Brain S.D. (2014). Calcitonin Gene-Related Peptide: Physiology and Pathophysiology. Physiol. Rev..

[38] Mai L., Liu Q., Huang F., He H., Fan W. (2021). Involvement of Mast Cells in the Pathophysiology of Pain. Front. Cell. Neurosci..

[39] Van Diest S.A., Stanisor O.I., Boeckxstaens G.E., De Jonge W.J., Van Den Wijngaard R.M. (2012). Relevance of Mast Cell–Nerve Interactions in Intestinal Nociception. Biochim. Biophys. Acta.

[40] Wouters M.M., Balemans D., Van Wanrooy S., Dooley J., Cibert-Goton V., Alpizar Y.A., Valdez-Morales E.E., Nasser Y., Van Veldhoven P.P., Vanbrabant W. (2016). Histamine Receptor H1–Mediated Sensitization of TRPV1 Mediates Visceral Hypersensitivity and Symptoms in Patients with Irritable Bowel Syndrome. Gastroenterology.

[41] Raffaelli B., Overeem L.H., Mecklenburg J., Hofacker M.D., Knoth H., Nowak C.P., Neeb L., Ebert A.D., Sehouli J., Mechsner S. (2021). Plasma Calcitonin Gene-related Peptide (CGRP) in Migraine and Endometriosis during the Menstrual Cycle. Ann. Clin. Transl. Neurol..

[42] Baethge C., Goldbeck-Wood S., Mertens S. (2019). SANRA: A Scale for the Quality Assessment of Narrative Review Articles. Res. Integr. Peer Rev..

[43] Symons L.K., Miller J.E., Kay V.R., Marks R.M., Liblik K., Koti M., Tayade C. (2018). The Immunopathophysiology of Endometriosis. Trends Mol. Med..

[44] Hogg C., Horne A.W., Greaves E. (2020). Endometriosis-Associated Macrophages: Origin, Phenotype, and Function. Front. Endocrinol..

[45] Forster R., Sarginson A., Velichkova A., Hogg C., Dorning A., Horne A.W., Saunders P.T.K., Greaves E. (2019). Macrophage-derived Insulin-like Growth Factor-1 Is a Key Neurotrophic and Nerve-sensitizing Factor in Pain Associated with Endometriosis. FASEB J..

[46] Zhu H., Wang Y., He Y., Yu W. (2022). Inflammation-Mediated Macrophage Polarization Induces TRPV1/TRPA1 Heteromers in Endometriosis. Am. J. Transl. Res..

[47] Kobayashi H., Imanaka S. (2022). Understanding the Molecular Mechanisms of Macrophage Polarization and Metabolic Reprogramming in Endometriosis: A Narrative Review. Reprod. Med. Biol..

[48] Crescenzi R., Donahue P.M.C., Petersen K.J., Garza M., Patel N., Lee C., Beckman J.A., Donahue M.J. (2020). Upper and Lower Extremity Measurement of Tissue Sodium and Fat Content in Patients with Lipedema. Obesity.

[49] Woolf C.J. (2011). Central Sensitization: Implications for the Diagnosis and Treatment of Pain. Pain.

[50] Morotti M., Vincent K., Brawn J., Zondervan K.T., Becker C.M. (2014). Peripheral Changes in Endometriosis-Associated Pain. Hum. Reprod. Update.

[51] Ping Z., Wen Z., Jinhua L., Jinghe L. (2019). Research on Central Sensitization of Endometriosis-Associated Pain: A Systematic Review of the Literature. J. Pain Res..

[52] Yan D., Liu X., Guo S.-W. (2019). Neuropeptides Substance P and Calcitonin Gene Related Peptide Accelerate the Development and Fibrogenesis of Endometriosis. Sci. Rep..

[53] Ren K., Dubner R. (2010). Interactions between the Immune and Nervous Systems in Pain. Nat. Med..

[54] As-Sanie S., Harris R.E., Napadow V., Kim J., Neshewat G., Kairys A., Williams D., Clauw D.J., Schmidt-Wilcke T. (2012). Changes in Regional Gray Matter Volume in Women with Chronic Pelvic Pain: A Voxel-Based Morphometry Study. Pain.

[55] Theoharides T.C., Tsilioni I., Bawazeer M. (2019). Mast Cells, Neuroinflammation and Pain in Fibromyalgia Syndrome. Front. Cell. Neurosci..

[56] Coxon L., Vollert J., Perro D., Lunde C.E., Ferreira-Gomes J., Charrua A., Abreu-Mendes P., Krassowski M., Birch J., Meijlink J. (2023). Comprehensive Quantitative Sensory Testing Shows Altered Sensory Function in Women with Chronic Pelvic Pain: Results from the Translational Research in Pelvic Pain (TRiPP) Study. Pain.

[57] Kosek E., Clauw D., Nijs J., Baron R., Gilron I., Harris R.E., Mico J.-A., Rice A.S.C., Sterling M. (2021). Chronic Nociplastic Pain Affecting the Musculoskeletal System: Clinical Criteria and Grading System. Pain.

[58] As-Sanie S., Kim J., Schmidt-Wilcke T., Sundgren P.C., Clauw D.J., Napadow V., Harris R.E. (2016). Functional Connectivity Is Associated with Altered Brain Chemistry in Women with Endometriosis-Associated Chronic Pelvic Pain. J. Pain.

[59] Michelini S., Chiurazzi P., Marino V., Dell’Orco D., Manara E., Baglivo M., Fiorentino A., Maltese P.E., Pinelli M., Herbst K.L. (2020). Aldo-Keto Reductase 1C1 (AKR1C1) as the First Mutated Gene in a Family with Nonsyndromic Primary Lipedema. Int. J. Mol. Sci..

[60] Song S.Y., Jung Y.W., Shin W., Park M., Lee G.W., Jeong S., An S., Kim K., Ko Y.B., Lee K.H. (2023). Endometriosis-Related Chronic Pelvic Pain. Biomedicines.

[61] Kruglikov I.L., Scherer P.E. (2024). Is the Endotoxin–Complement Cascade the Major Driver in Lipedema?. Trends Endocrinol. Metab..

[62] Muddam M.R., Obajeun O.A., Abaza A., Jaramillo A.P., Sid Idris F., Anis Shaikh H., Vahora I., Moparthi K.P., Al Rushaidi M.T., Nath T.S. (2023). Efficacy and Safety of Anti-Calcitonin Gene-Related Peptide (CGRP) Monoclonal Antibodies in Preventing Migraines: A Systematic Review. Cureus.

